# 1-[(Phenyl­iminio)amino]-2-naphtho­late

**DOI:** 10.1107/S1600536810023329

**Published:** 2010-06-23

**Authors:** Ji-Jun Xu, Jun Li, Min Pi, Chuan-Ming Jin

**Affiliations:** a Hubei Key Laboratory of Pollutant Analysis & Reuse Technology, College of Chemistry and Environmental Engineering, Hubei Normal University, Huangshi, Hubei 435002, People’s Republic of China

## Abstract

In the zwitterionic title compound, C_16_H_12_N_2_O, the dihedral angle between the benzene ring and naphthalene ring system is 2.0 (1)°. The azo group adopts a *trans* configuration and an intra­molecular N—H⋯O hydrogen bond is found. In the crystal, the mol­ecules are packed by strong π–π inter­actions [centroid–centroid distance between aromatic rings = 3.375 (3) Å].

## Related literature

For general background to the use of azo compounds as dyes, pigments and advanced materials, see: Lee *et al.* (2004 ▶); Oueslati *et al.* (2004 ▶). Many azo compounds have been synthesized by diazo­tization and diazo coupling reactions, see: Wang *et al.* (2003 ▶).
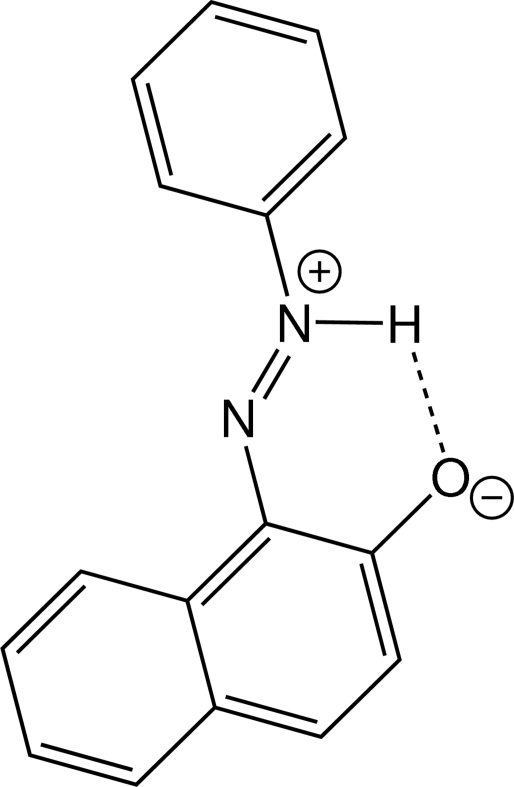


## Experimental

### 

#### Crystal data

C_16_H_12_N_2_O
*M* *_r_* = 248.28
Monoclinic, 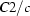

*a* = 27.8713 (4) Å
*b* = 6.0248 (1) Å
*c* = 14.9199 (2) Å
β = 103.570 (2)°
*V* = 2435.40 (7) Å^3^
*Z* = 8
Mo *K*α radiation
μ = 0.09 mm^−1^
*T* = 200 K
0.13 × 0.10 × 0.08 mm

#### Data collection

Bruker SMART APEX CCD area-detector diffractometer
Absorption correction: multi-scan (*SADABS*; Sheldrick, 1996 ▶) *T*_min_ = 0.989, *T*_max_ = 0.993
8859 measured reflections
3002 independent reflections
2536 reflections with *I* > 2σ(*I*)
*R*_int_ = 0.088

#### Refinement

*R*[*F* ^2^ > 2σ(*F* ^2^)] = 0.063
*wR*(*F* ^2^) = 0.169
*S* = 1.08
3002 reflections
175 parameters
H atoms treated by a mixture of independent and constrained refinement
Δρ_max_ = 0.48 e Å^−3^
Δρ_min_ = −0.23 e Å^−3^

### 

Data collection: *SMART* (Bruker, 2001 ▶); cell refinement: *SAINT-Plus* (Bruker, 2001 ▶); data reduction: *SAINT-Plus*; program(s) used to solve structure: *SHELXS97* (Sheldrick, 2008 ▶); program(s) used to refine structure: *SHELXL97* (Sheldrick, 2008 ▶); molecular graphics: *SHELXTL* (Sheldrick, 2008 ▶); software used to prepare material for publication: *SHELXTL*.

## Supplementary Material

Crystal structure: contains datablocks I, global. DOI: 10.1107/S1600536810023329/rk2210sup1.cif

Structure factors: contains datablocks I. DOI: 10.1107/S1600536810023329/rk2210Isup2.hkl

Additional supplementary materials:  crystallographic information; 3D view; checkCIF report

## Figures and Tables

**Table 1:** Hydrogen-bond geometry (Å, °)

*D*—H⋯*A*	*D*—H	H⋯*A*	*D*⋯*A*	*D*—H⋯*A*
N2—H2*A*⋯O1	0.895 (19)	1.803 (18)	2.5545 (17)	140.0 (16)

**Fig. 1.**
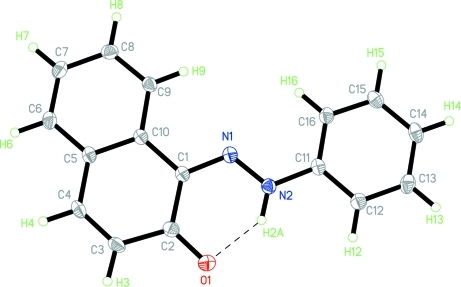
The structure of title compound showing the atom–numbering scheme. Displacement ellipsoids are drawn at the 30% probability level. H atoms are presented as a small spheres of arbitrary radius.

## supplementary crystallographic information

### Comment

Azo-compounds are very important in the fields of dyes, pigments and advanced materials (Lee *et al.*, 2004; Oueslati *et al.*, 2004). Azo-dyes are synthetic pigments that contain an azo-group, as part of the structure. Azo-groups do not occur naturally. Many azo-compounds have been synthesized by the diazotization and diazo coupling reaction (Wang *et al.*, 2003). The title compound, I, was obtained through the diazotization of aniline followed by a coupling reaction with 2-naphthol.

The molecular structure of I is illustrated in Fig. 1. The molecule adopts an *anti*–configuration with the two aryl groups reside on the opposite side of azo–group. The dihedral angle between the benzene ring and naphthalene ring is 2.0 (1)°. An intramolecular N—H···O hydrogen bond is found (Table 1). It is more interesting, that hydrogen atom in the OH-group has transfer to N atom in the azo-group to form the structure of dipolar ion. Moreover, different Fourier map indicate hydrogen site location is closer to nitrogen atom of azo-group. In the crystal molecules are packed by the weak π–π interactions with the closest approach between centroids of aromatic rings is 3.375 (3)Å.

### Experimental

The title compound was prepared by a similar method of other aromatic azo–compounds (Wang *et al.*, 2003). Single crystals of I were obtained by slow evaporation from a petroleum ether ethyl acetate (2/1 *v*/*v*) solution system.

### Refinement

The H atoms based on C atoms were positioned geometrically at the distance of 0.95Å, and refined in a riding model with *U*_iso_(H) = 1.2*U*_eq_(C). The H atom of amino-group was refined freely.

### Crystal data

**Table:**

C_16_H_12_N_2_O	*F*(000) = 1040
*M**_r_* = 248.28	*D*_x_ = 1.354 Mg m^−^^3^
Monoclinic, *C*2/*c*	Mo *K*α radiation, λ = 0.71073 Å
Hall symbol: -C 2yc	Cell parameters from 2783 reflections
*a* = 27.8713 (4) Å	θ = 2.8–28.2°
*b* = 6.0248 (1) Å	µ = 0.09 mm^−^^1^
*c* = 14.9199 (2) Å	*T* = 200 K
β = 103.570 (2)°	Block, red
*V* = 2435.40 (7) Å^3^	0.13 × 0.10 × 0.08 mm
*Z* = 8	

### Data collection

**Table:**

Bruker SMART APEX CCD area-detector diffractometer	3002 independent reflections
Radiation source: fine–focus sealed tube	2536 reflections with *I* > 2σ(*I*)
graphite	*R*_int_ = 0.088
φ and ω scans	θ_max_ = 28.3°, θ_min_ = 2.8°
Absorption correction: multi-scan (*SADABS*; Sheldrick, 1996)	*h* = −35→36
*T*_min_ = 0.989, *T*_max_ = 0.993	*k* = −8→8
8859 measured reflections	*l* = −15→19

### Refinement

**Table:**

Refinement on *F*^2^	Primary atom site location: structure-invariant direct methods
Least-squares matrix: full	Secondary atom site location: difference Fourier map
*R*[*F*^2^ > 2σ(*F*^2^)] = 0.063	Hydrogen site location: inferred from neighbouring sites
*wR*(*F*^2^) = 0.169	H atoms treated by a mixture of independent and constrained refinement
*S* = 1.08	*w* = 1/[σ^2^(*F*_o_^2^) + (0.0799*P*)^2^ + 1.0846*P*] where *P* = (*F*_o_^2^ + 2*F*_c_^2^)/3
3002 reflections	(Δ/σ)_max_ = 0.001
175 parameters	Δρ_max_ = 0.48 e Å^−^^3^
0 restraints	Δρ_min_ = −0.23 e Å^−^^3^

### Special details

**Table:**

Geometry. All s.u.'s (except the s.u. in the dihedral angle between two l.s. planes) are estimated using the full covariance matrix. The cell s.u.'s are taken into account individually in the estimation of s.u.'s in distances, angles and torsion angles; correlations between s.u.'s in cell parameters are only used when they are defined by crystal symmetry. An approximate (isotropic) treatment of cell s.u.'s is used for estimating s.u.'s involving l.s. planes.
Refinement. Refinement of *F*^2^ against ALL reflections. The weighted *R*–factor *wR* and goodness of fit *S* are based on *F*^2^, conventional *R*–factors *R* are based on *F*, with *F* set to zero for negative *F*^2^. The threshold expression of *F*^2^ > σ(*F*^2^) is used only for calculating *R*–factors(gt) *etc*. and is not relevant to the choice of reflections for refinement. *R*–factors based on *F*^2^ are statistically about twice as large as those based on *F*, and *R*–factors based on ALL data will be even larger.

### Fractional atomic coordinates and isotropic or equivalent isotropic displacement parameters (Å^2^)

**Table:**

	*x*	*y*	*z*	*U*_iso_*/*U*_eq_	
C1	0.14696 (5)	0.0629 (2)	0.09018 (10)	0.0244 (3)	
C2	0.19947 (5)	0.0124 (3)	0.11604 (10)	0.0287 (3)	
C3	0.21439 (6)	−0.1846 (3)	0.17062 (11)	0.0330 (4)	
H3	0.2485	−0.2197	0.1901	0.040*	
C4	0.18102 (6)	−0.3195 (3)	0.19449 (11)	0.0319 (4)	
H4	0.1924	−0.4484	0.2298	0.038*	
C5	0.12895 (5)	−0.2771 (2)	0.16900 (10)	0.0256 (3)	
C6	0.09522 (6)	−0.4260 (3)	0.19251 (11)	0.0314 (4)	
H6	0.1070	−0.5575	0.2256	0.038*	
C7	0.04557 (6)	−0.3840 (3)	0.16840 (11)	0.0337 (4)	
H7	0.0230	−0.4870	0.1839	0.040*	
C8	0.02823 (6)	−0.1896 (3)	0.12098 (11)	0.0324 (4)	
H8	−0.0062	−0.1594	0.1052	0.039*	
C9	0.06067 (5)	−0.0409 (2)	0.09676 (10)	0.0282 (3)	
H9	0.0484	0.0912	0.0648	0.034*	
C10	0.11155 (5)	−0.0826 (2)	0.11877 (9)	0.0237 (3)	
C11	0.13828 (6)	0.5505 (2)	−0.04777 (9)	0.0255 (3)	
C12	0.17170 (6)	0.6926 (3)	−0.07488 (11)	0.0311 (4)	
H12	0.2061	0.6623	−0.0576	0.037*	
C13	0.15433 (7)	0.8788 (3)	−0.12732 (11)	0.0355 (4)	
H13	0.1769	0.9765	−0.1460	0.043*	
C14	0.10432 (6)	0.9226 (3)	−0.15249 (11)	0.0340 (4)	
H14	0.0925	1.0507	−0.1879	0.041*	
C15	0.07150 (6)	0.7790 (3)	−0.12588 (11)	0.0329 (4)	
H15	0.0371	0.8088	−0.1438	0.039*	
C16	0.08806 (6)	0.5922 (2)	−0.07338 (11)	0.0292 (3)	
H16	0.0653	0.4944	−0.0553	0.035*	
N1	0.12797 (5)	0.23592 (19)	0.03768 (8)	0.0253 (3)	
N2	0.15775 (5)	0.3675 (2)	0.00730 (9)	0.0268 (3)	
H2A	0.1899 (7)	0.333 (3)	0.0227 (13)	0.032*	
O1	0.23107 (4)	0.1341 (2)	0.09144 (9)	0.0387 (3)	

### Atomic displacement parameters (Å^2^)

**Table:**

	*U*^11^	*U*^22^	*U*^33^	*U*^12^	*U*^13^	*U*^23^
C1	0.0285 (7)	0.0253 (7)	0.0190 (7)	0.0024 (5)	0.0050 (5)	−0.0008 (5)
C2	0.0281 (7)	0.0323 (8)	0.0243 (7)	0.0009 (6)	0.0030 (6)	−0.0012 (6)
C3	0.0263 (7)	0.0406 (9)	0.0288 (8)	0.0067 (6)	−0.0003 (6)	0.0043 (7)
C4	0.0357 (8)	0.0321 (8)	0.0255 (8)	0.0084 (6)	0.0020 (6)	0.0068 (6)
C5	0.0327 (8)	0.0274 (7)	0.0166 (7)	0.0027 (6)	0.0058 (6)	−0.0008 (5)
C6	0.0416 (9)	0.0292 (7)	0.0250 (8)	0.0036 (6)	0.0110 (6)	0.0037 (6)
C7	0.0381 (9)	0.0335 (8)	0.0329 (9)	−0.0032 (6)	0.0151 (7)	0.0020 (6)
C8	0.0292 (8)	0.0388 (9)	0.0306 (8)	0.0029 (6)	0.0097 (6)	0.0001 (6)
C9	0.0300 (7)	0.0307 (7)	0.0241 (7)	0.0059 (6)	0.0069 (6)	0.0034 (6)
C10	0.0292 (7)	0.0258 (7)	0.0163 (6)	0.0032 (5)	0.0059 (5)	−0.0013 (5)
C11	0.0351 (8)	0.0239 (7)	0.0184 (7)	0.0026 (6)	0.0080 (6)	−0.0009 (5)
C12	0.0343 (8)	0.0324 (8)	0.0284 (8)	0.0010 (6)	0.0113 (6)	0.0007 (6)
C13	0.0489 (10)	0.0314 (8)	0.0304 (8)	−0.0022 (7)	0.0180 (7)	0.0031 (6)
C14	0.0521 (10)	0.0271 (7)	0.0232 (8)	0.0076 (7)	0.0100 (7)	0.0030 (6)
C15	0.0387 (8)	0.0315 (8)	0.0274 (8)	0.0071 (6)	0.0057 (7)	−0.0008 (6)
C16	0.0340 (8)	0.0273 (7)	0.0276 (8)	0.0001 (6)	0.0101 (6)	0.0001 (6)
N1	0.0317 (7)	0.0257 (6)	0.0192 (6)	0.0011 (5)	0.0073 (5)	−0.0017 (4)
N2	0.0284 (6)	0.0271 (6)	0.0253 (7)	0.0019 (5)	0.0071 (5)	0.0023 (5)
O1	0.0284 (6)	0.0426 (7)	0.0437 (7)	−0.0017 (5)	0.0058 (5)	0.0084 (5)

### Geometric parameters (Å, °)

**Table:**

C1—N1	1.3364 (18)	C9—C10	1.401 (2)
C1—C2	1.455 (2)	C9—H9	0.9500
C1—C10	1.457 (2)	C11—C16	1.385 (2)
C2—O1	1.2650 (18)	C11—C12	1.393 (2)
C2—C3	1.444 (2)	C11—N2	1.4058 (18)
C3—C4	1.344 (2)	C12—C13	1.389 (2)
C3—H3	0.9500	C12—H12	0.9500
C4—C5	1.434 (2)	C13—C14	1.381 (2)
C4—H4	0.9500	C13—H13	0.9500
C5—C6	1.402 (2)	C14—C15	1.383 (2)
C5—C10	1.4141 (19)	C14—H14	0.9500
C6—C7	1.369 (2)	C15—C16	1.387 (2)
C6—H6	0.9500	C15—H15	0.9500
C7—C8	1.395 (2)	C16—H16	0.9500
C7—H7	0.9500	N1—N2	1.3033 (17)
C8—C9	1.380 (2)	N2—H2A	0.895 (19)
C8—H8	0.9500		
			
N1—C1—C2	123.63 (13)	C10—C9—H9	119.6
N1—C1—C10	116.02 (13)	C9—C10—C5	118.37 (13)
C2—C1—C10	120.32 (13)	C9—C10—C1	122.79 (13)
O1—C2—C3	120.81 (14)	C5—C10—C1	118.82 (13)
O1—C2—C1	121.82 (13)	C16—C11—C12	120.65 (13)
C3—C2—C1	117.37 (13)	C16—C11—N2	122.01 (13)
C4—C3—C2	121.37 (14)	C12—C11—N2	117.33 (14)
C4—C3—H3	119.3	C13—C12—C11	119.46 (15)
C2—C3—H3	119.3	C13—C12—H12	120.3
C3—C4—C5	122.82 (14)	C11—C12—H12	120.3
C3—C4—H4	118.6	C14—C13—C12	120.23 (15)
C5—C4—H4	118.6	C14—C13—H13	119.9
C6—C5—C10	119.73 (14)	C12—C13—H13	119.9
C6—C5—C4	121.06 (13)	C13—C14—C15	119.73 (14)
C10—C5—C4	119.21 (13)	C13—C14—H14	120.1
C7—C6—C5	120.78 (14)	C15—C14—H14	120.1
C7—C6—H6	119.6	C14—C15—C16	120.98 (15)
C5—C6—H6	119.6	C14—C15—H15	119.5
C6—C7—C8	119.79 (14)	C16—C15—H15	119.5
C6—C7—H7	120.1	C11—C16—C15	118.95 (14)
C8—C7—H7	120.1	C11—C16—H16	120.5
C9—C8—C7	120.52 (14)	C15—C16—H16	120.5
C9—C8—H8	119.7	N2—N1—C1	118.77 (12)
C7—C8—H8	119.7	N1—N2—C11	119.36 (13)
C8—C9—C10	120.77 (14)	N1—N2—H2A	116.7 (11)
C8—C9—H9	119.6	C11—N2—H2A	123.9 (11)
			
N1—C1—C2—O1	1.5 (2)	C4—C5—C10—C1	3.3 (2)
C10—C1—C2—O1	179.23 (13)	N1—C1—C10—C9	−2.8 (2)
N1—C1—C2—C3	−177.99 (13)	C2—C1—C10—C9	179.28 (13)
C10—C1—C2—C3	−0.2 (2)	N1—C1—C10—C5	175.65 (12)
O1—C2—C3—C4	−177.66 (15)	C2—C1—C10—C5	−2.3 (2)
C1—C2—C3—C4	1.8 (2)	C16—C11—C12—C13	0.6 (2)
C2—C3—C4—C5	−0.8 (2)	N2—C11—C12—C13	−178.35 (13)
C3—C4—C5—C6	177.74 (15)	C11—C12—C13—C14	−0.1 (2)
C3—C4—C5—C10	−1.8 (2)	C12—C13—C14—C15	−0.5 (2)
C10—C5—C6—C7	−0.8 (2)	C13—C14—C15—C16	0.6 (2)
C4—C5—C6—C7	179.65 (15)	C12—C11—C16—C15	−0.6 (2)
C5—C6—C7—C8	−0.9 (2)	N2—C11—C16—C15	178.39 (13)
C6—C7—C8—C9	1.1 (2)	C14—C15—C16—C11	−0.1 (2)
C7—C8—C9—C10	0.4 (2)	C2—C1—N1—N2	0.2 (2)
C8—C9—C10—C5	−2.0 (2)	C10—C1—N1—N2	−177.61 (12)
C8—C9—C10—C1	176.43 (13)	C1—N1—N2—C11	−179.81 (12)
C6—C5—C10—C9	2.2 (2)	C16—C11—N2—N1	−2.1 (2)
C4—C5—C10—C9	−178.23 (13)	C12—C11—N2—N1	176.92 (12)
C6—C5—C10—C1	−176.27 (13)		

### Hydrogen-bond geometry (Å, °)

**Table:**

*D*—H···*A*	*D*—H	H···*A*	*D*···*A*	*D*—H···*A*
N2—H2A···O1	0.895 (19)	1.803 (18)	2.5545 (17)	140.0 (16)
